# Supplementary figures and images for: Consistent and reproducible long-term in vitro growth of health and disease-associated oral subgingival biofilms
Source: BMC Microbiol. 2018 Jul 11;18:70. doi: 10.1186/s12866-018-1212-x (PMC6042318; doi:10.1186/s12866-018-1212-x)

**A**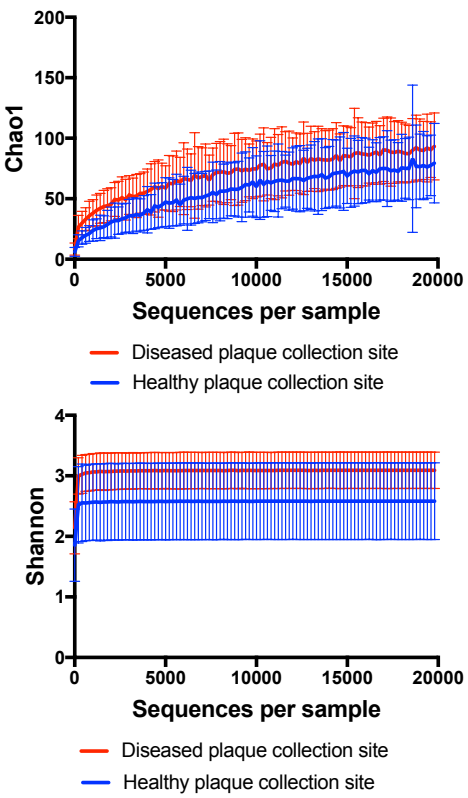**B**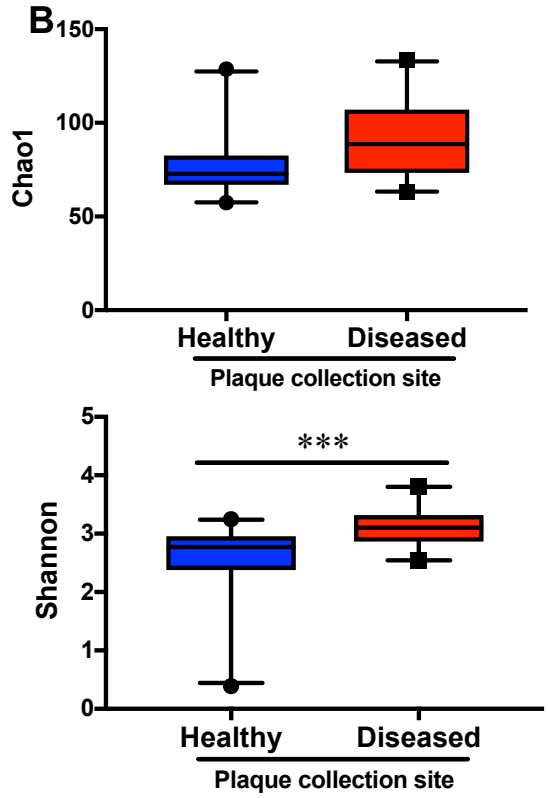**C**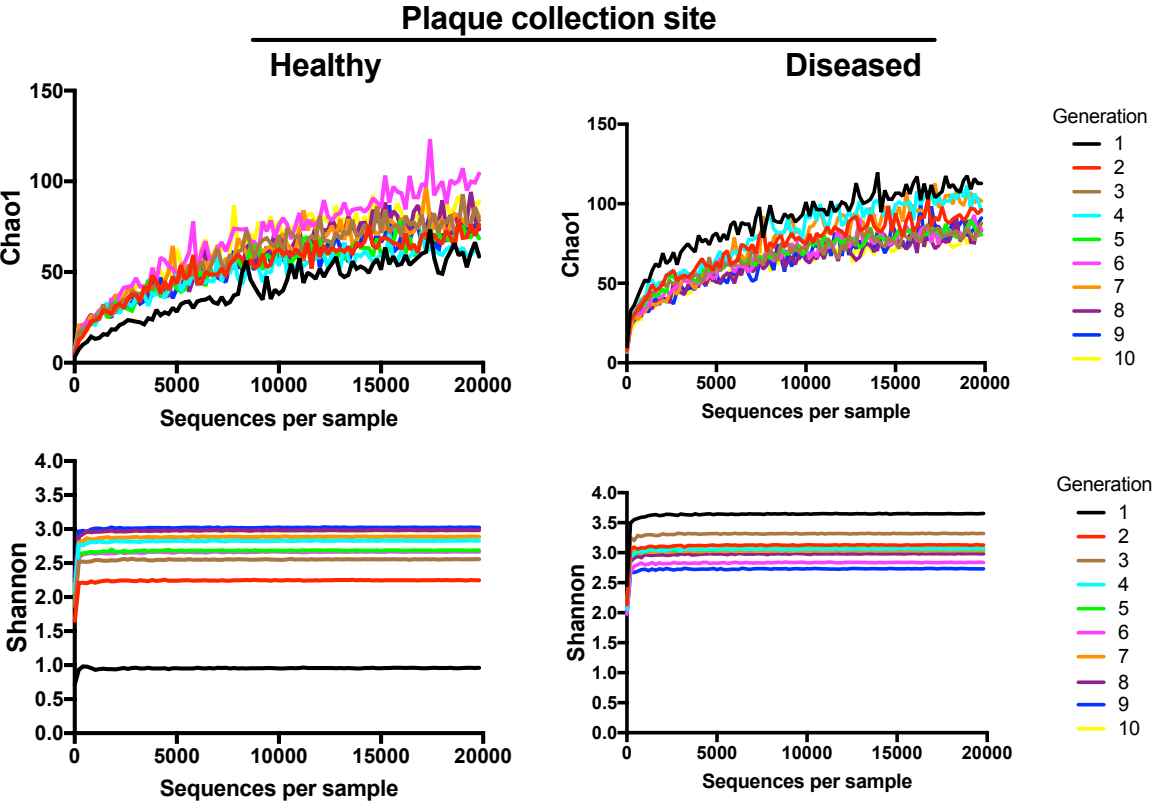

Supplement: Supplementary file 3 — Figure S1. Alpha diversity of healthy and disease site-derived biofilms. (a) Rarefaction curves of Chao1 index (top) and Shannon index (bottom) of all combined generations for healthy and disease site-derived biofilms. (b) Chao1 index (top) and Shannon index (bottom) are significantly different between healthy and disease site-derived biofilms at 20000 reads. (c) Rarefaction curves of Chao1 index (top) and Shannon index (bottom) of healthy (left) and disease (right) site-derived biofilms at each generation. Box plots show 5–95 percentile. All error bars indicate ± SD. (PDF 78 kb) [file 12866_2018_1212_MOESM3_ESM.pdf]

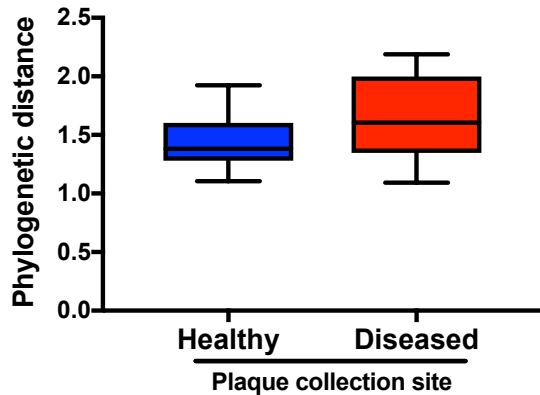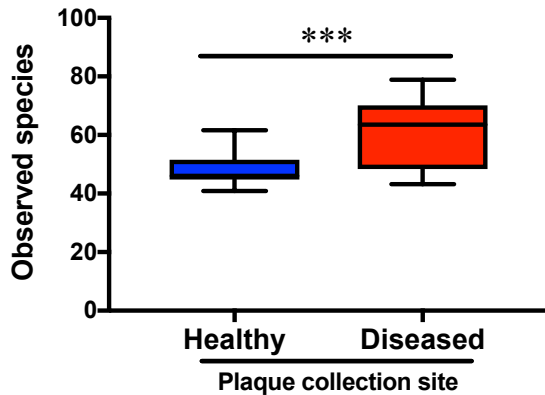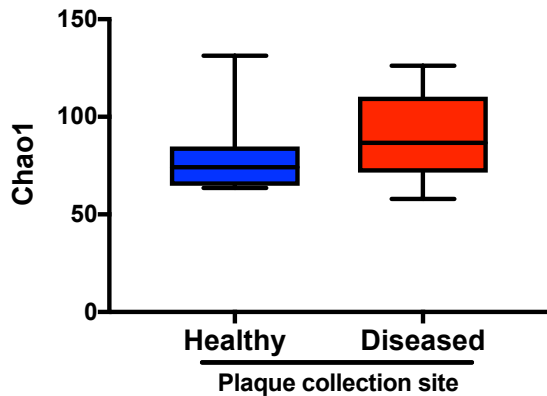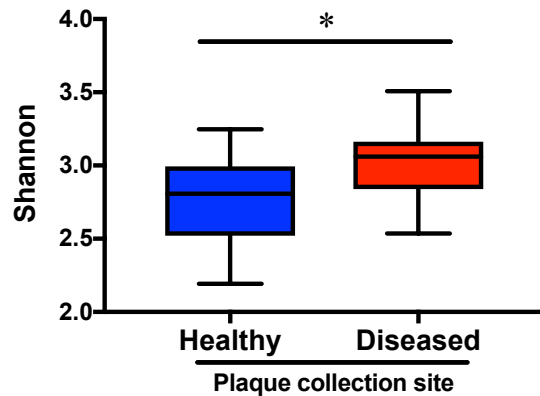

Supplement: Supplementary file 4 — Figure S2. Alpha diversity of healthy and disease-site derived biofilms generations 2–10. Alpha diversity score for Faith’s phylogenetic distance, Observed species, Chao1 and Shannon indexes at 20000 reads averaged across generations 2–10. Box plots show 5–95 percentile. All error bars indicate ± SD. * p < 0.05, *** p < 0.001. (PDF 26 kb) [file 12866_2018_1212_MOESM4_ESM.pdf]

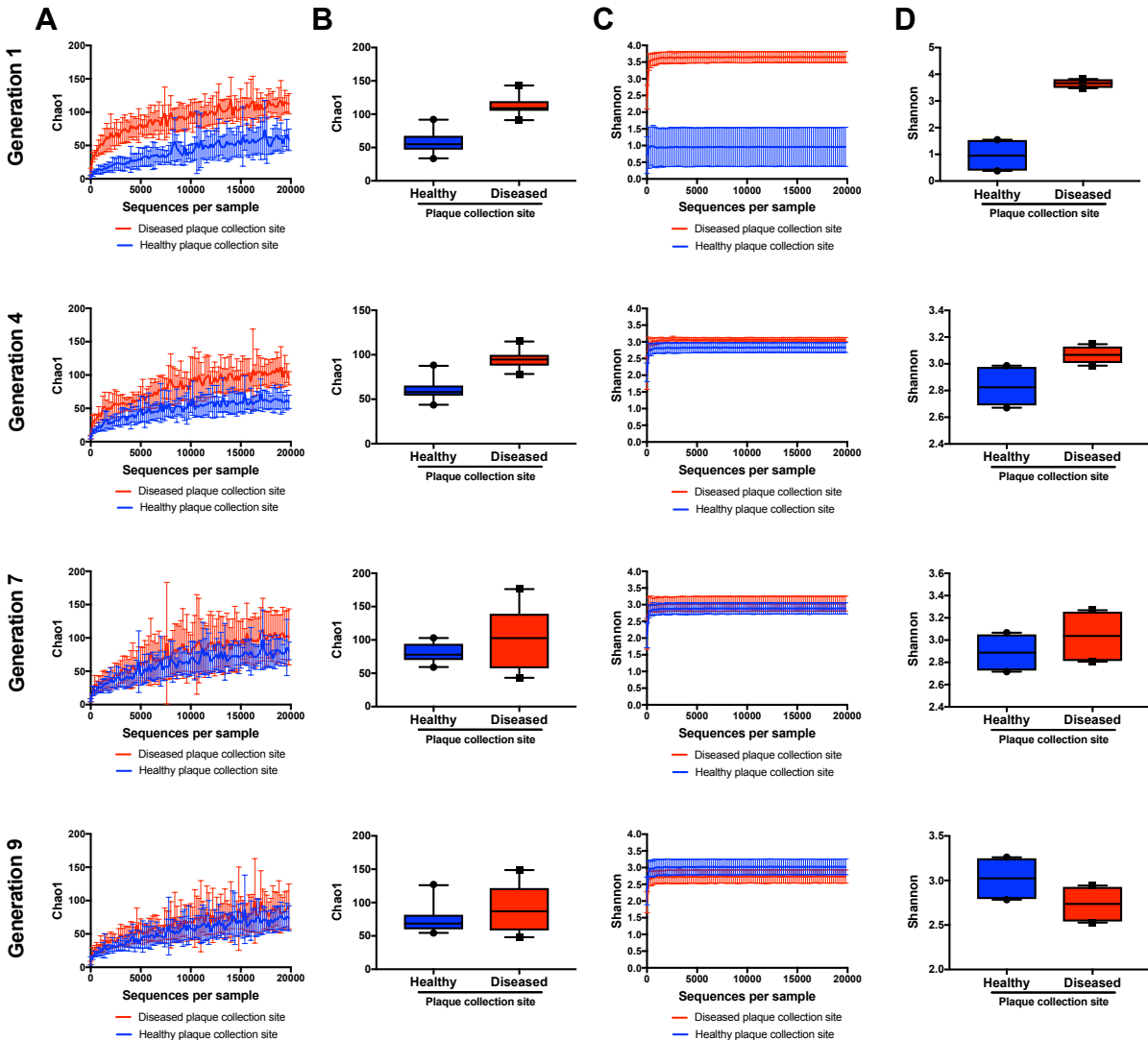

Supplement: Supplementary file 5 — Figure S3. Alpha diversity of healthy and disease site-derived biofilms throughout the generations. (a) Chao1 index rarefaction curves at generations 1, 4, 7, and 9. (b) Chao1 index of biofilms by generation at 20000 reads. (c) Shannon index rarefaction curves at generations 1, 4, 7, and 9. (d) Shannon index in biofilms by generation at 20000 reads. N = 2 biofilms per group so statistical significance could not be calculated. Box plots show 5–95 percentile. All error bars indicate ± SD. (PDF 103 kb) [file 12866_2018_1212_MOESM5_ESM.pdf]

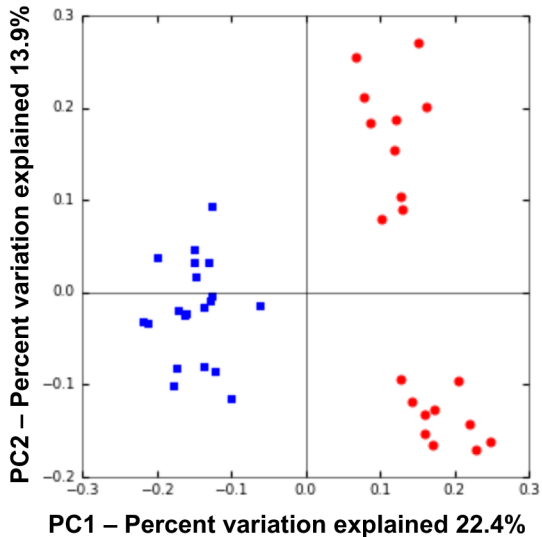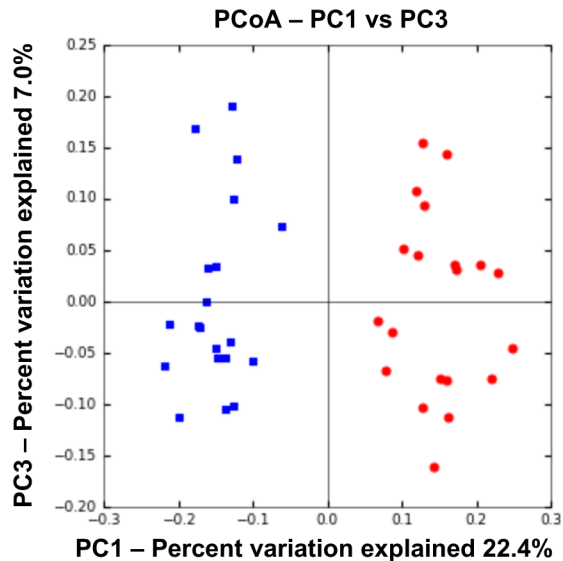

● Diseased plaque collection site

■ Healthy plaque collection site

Supplement: Supplementary file 6 — Figure S4. Principal coordinates analysis of beta diversity by Sorensen distance of healthy and disease site-derived biofilms. Biofilms cluster based on disease status of original plaque inocula and group separation is significant based on adonis analysis (p < 0.001). Additionally, disease site-derived biofilms cluster into 2 groups based on plaque inocula donor. (PDF 5954 kb) [file 12866_2018_1212_MOESM6_ESM.pdf]

**A**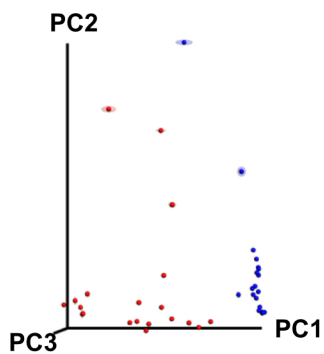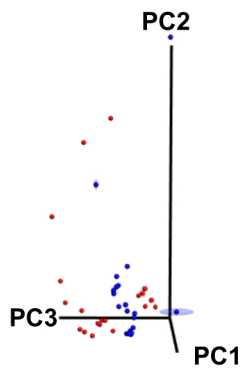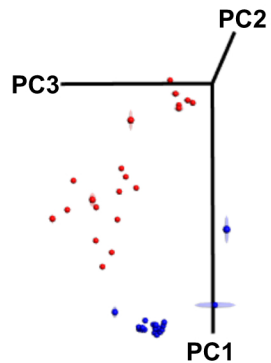**B**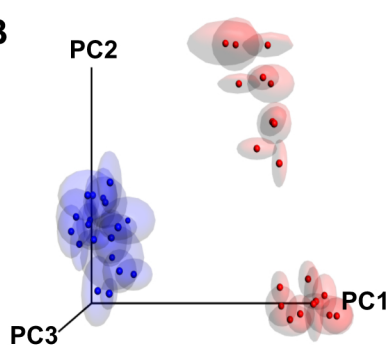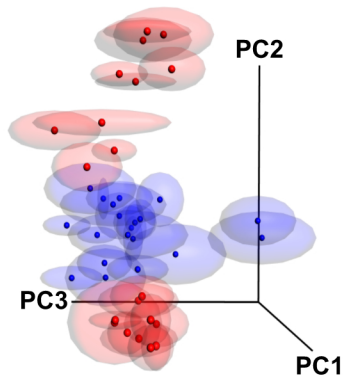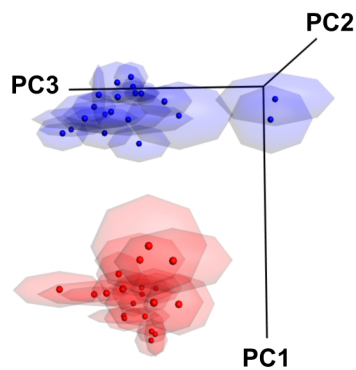

Supplement: Supplementary file 7 — Figure S5. Principal coordinates analysis of jack-knifed beta diversity by weighted UniFrac and Sorensen distances of biofilms. Biofilms cluster based on disease status of original plaque inocula and disease site-derived biofilms separate based on donor. (a) Biofilm coordinates exhibit very little variation in PCoA based on weighted UniFrac distance. (b) Biofilm coordinates demonstrate variation in PCoA based on Sorensen distance but retain distinct clustering of healthy and disease site-derived groups. Additionally, disease site-derived biofilms likewise retain distinct separation into groups based on plaque inocula donor. (PDF 1518 kb) [file 12866_2018_1212_MOESM7_ESM.pdf]

**A**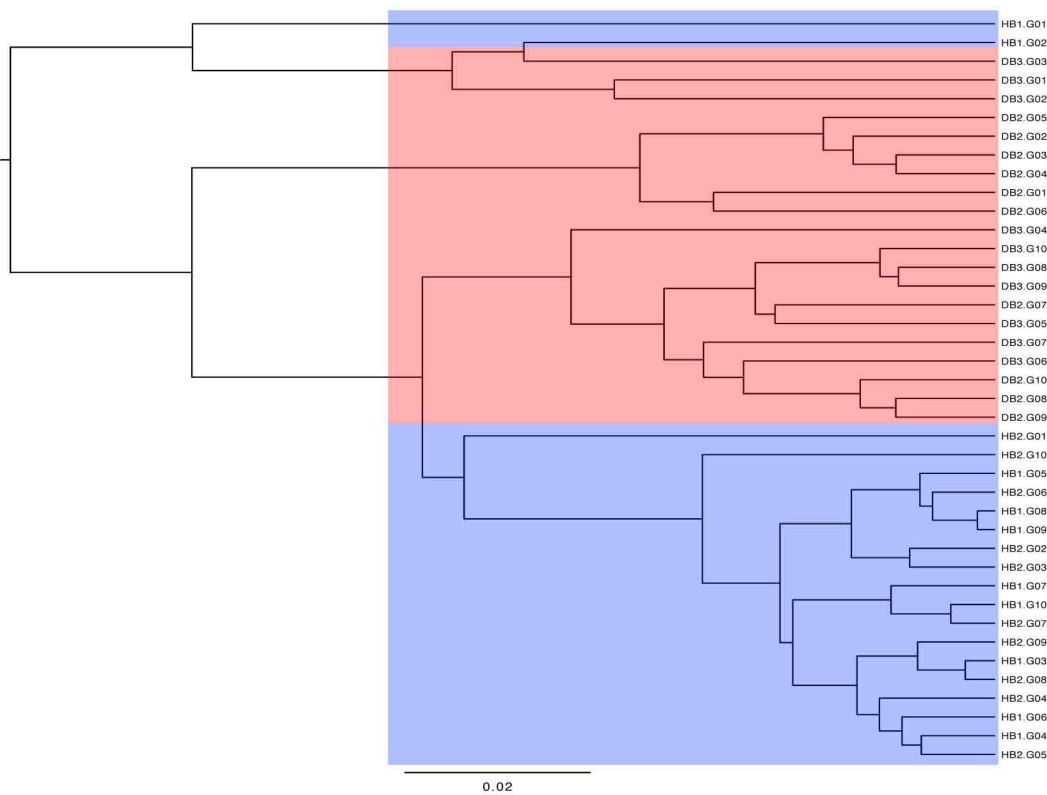**B**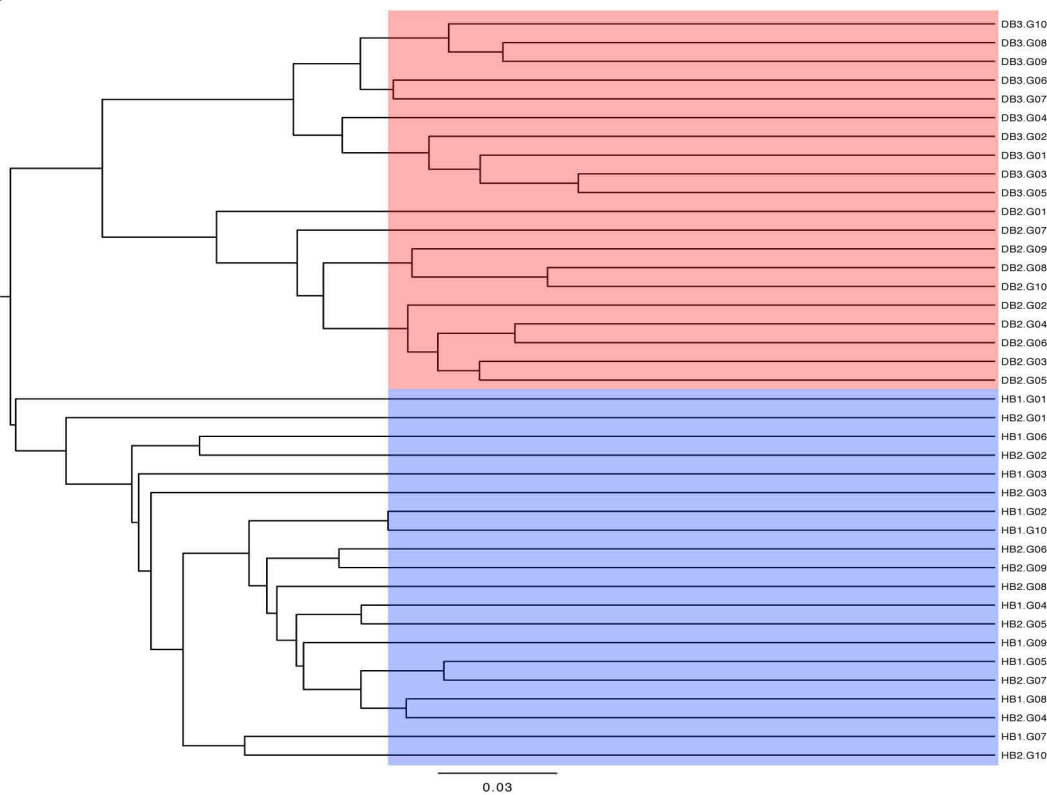

Supplement: Supplementary file 8 — Figure S6. UPGMA consensus trees of jackknifed beta-diversity measurements. (a) Weighted UniFrac distance groups several early biofilm generations despite differences in plaque source, but otherwise distinguishes between healthy and disease site-derived biofilm lineages. (b) Sorensen distance clearly distinguished between healthy and disease site-derived biofilm lineages. (PDF 532 kb) [file 12866_2018_1212_MOESM8_ESM.pdf]

■ Diseased plaque collection site 
 ■ Healthy plaque collection site 
 95% confidence intervals

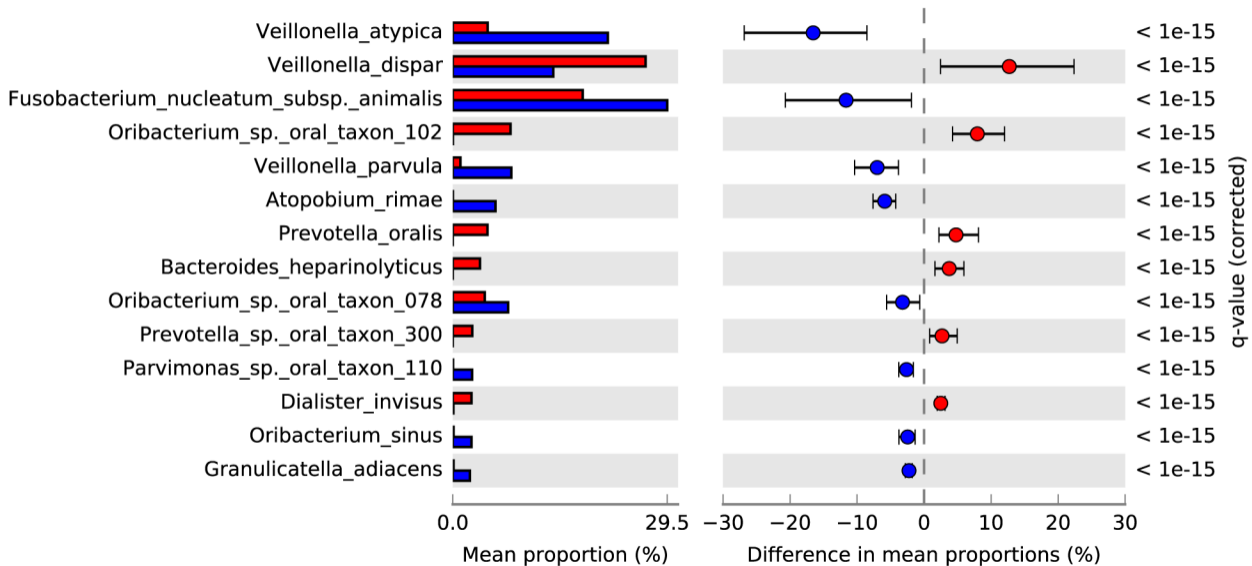

Supplement: Supplementary file 9 — Figure S7. Differential abundance of species present at > 0.1% abundance between healthy and disease site-derived biofilms. Species statistically different (q ≤ 0.05) and with an effect size (DP) > 1 by analysis in STAMP are considered differentially abundant. (PDF 5946 kb) [file 12866_2018_1212_MOESM9_ESM.pdf]
